# Online versus in-person comparison of Microscale Audit of Pedestrian Streetscapes (MAPS) assessments: reliability of alternate methods

**DOI:** 10.1186/s12942-017-0101-0

**Published:** 2017-08-04

**Authors:** Christine B. Phillips, Jessa K. Engelberg, Carrie M. Geremia, Wenfei Zhu, Jonathan M. Kurka, Kelli L. Cain, James F. Sallis, Terry L. Conway, Marc A. Adams

**Affiliations:** 1 School of Nutrition and Health Promotion, Arizona State University, Phoenix, AZ USA; 2 Department of Family and Preventive Medicine, University of California, San Diego, San Diego, CA USA; 3 School of Physical Education, Shaanxi Normal University, Xi’an, Shaanxi China

**Keywords:** Built environment, Walkability, Direct observation, Measurement, Physical activity, Walking, Virtual observation

## Abstract

**Background:**

An online version of the Microscale Audit of Pedestrian Streetscapes (Abbreviated) tool was adapted to virtually audit built environment features supportive of physical activity. The current study assessed inter-rater reliability of MAPS Online between in-person raters and online raters unfamiliar with the regions.

**Methods:**

In-person and online audits were conducted for a total of 120 quarter-mile routes (60 per site) in Phoenix, AZ and San Diego, CA. Routes in each city included 40 residential origins stratified by walkability and SES, and 20 commercial centers. In-person audits were conducted by raters residing in their region. Online audits were conducted by raters in the alternate location using Google Maps (Aerial and Street View) images. The MAPS Abbreviated Online tool consisted of four sections: overall route, street segments, crossings and cul-de-sacs. Items within each section were grouped into subscales, and inter-rater reliability (ICCs) was assessed for subscales at multiple levels of aggregation.

**Results:**

Online and in-person audits showed excellent agreement for overall positive microscale (ICC = 0.86, 95% CI [0.80, 0.90]) and grand scores (ICC = 0.93, 95% CI [0.89, 0.95]). Substantial to near-perfect agreement was found for 21 of 30 (70%) subscales, valence, and subsection scores, with ICCs ranging from 0.62, 95% CI [0.50, 0.72] to 0.95, 95% CI [0.93, 0.97]. Lowest agreement was found for the aesthetics and social characteristics scores, with ICCs ranging from 0.07, 95% CI [−0.12, 0.24] to 0.27, 95% CI [0.10, 0.43].

**Conclusions:**

Results support use of the MAPS Abbreviated Online tool to reliably assess microscale neighborhood features that support physical activity and may be used by raters residing in different geographic regions and unfamiliar with the audit areas.

## Background

Inherent to ecological models is a tenet that the environment affects health behaviors [1]. Supportive features of the built environment may be particularly relevant to physical activity [2–5] and often result in health, social and economic co-benefits [5]. The built environment is frequently characterized by macro-level attributes such as walkability, street connectivity or population density. However, *microscale* level details may be important [6–8]. For example, the presence, quality, designs and features of sidewalks, streets, intersections (e.g., sidewalk buffers, transit stops, crosswalk amenities), and streetscape aesthetics and social characteristics (e.g., public art, landscape upkeep, broken windows, graffiti) may help explain physical activity. Modifying microscale features to support health behaviors is easier and less costly than modifying macro-level features, and may provide significant public health returns relative to resource investment.

### In-person microscale assessments

Several field audit tools have been developed to evaluate microscale features for active living [8–10]. Evidence from field audits suggests that microscale neighborhood characteristics strongly relate to physical activity across the life span [3], even after controlling for macro-level walkability [3]. However, detailed microscale data obtained from in-person audits comes at a high price of time and monetary resources. For example, in-person audits require extensive training of staff, driving time and travel costs, and sometimes lodging costs to assess areas outside of one’s city. In-person audits are vulnerable to unfavorable weather conditions and may present potential safety risks for auditors. These elements are likely impediments to advancing research and practical implementation. Thus, a relatively small body of work has examined the relationships between microscale features and physical activity, and studies have generally been constricted in the number and/or size of geographic areas studied [2, 3, 8, 11, 12]. The global consequence of a limited research base and relatively few geographic areas is a lack of understanding about how microscale features in communities throughout the world influence physical activity behaviors.

### Online audits

“Virtual audits” address many of the limitations of in-person audits. Virtual audit methods have been developed using widely-available online satellite imagery or omni-directional imagery such as Bing Maps Streetside or Google Maps Street View, to conduct virtual microscale audits. Findings have been mostly promising, with virtual audits largely corresponding to in-person direct observations [13–23]. Though encouraging, the generalizability of existing studies is limited, and several studies were constrained to a single city or had small sample sizes [13, 15–17, 20, 24].

One potential benefit of virtual audits is the ability to conduct large-scale studies encompassing diverse geographic areas. Any geographic area accessible via online mapping services could be audited from a remote location. However, one concern is that centralized raters’ interpretations of virtual imagery may differ according to degree of contextual understanding or familiarity with an area [25, 26]. Most previous studies either did not specify the location of virtual raters or used raters from the audit area, creating uncertainty about whether raters’ familiarity with an area influenced rater agreement. Only two studies have addressed the issue of area-familiarity between raters using virtual microscale audit tools. Zhu and colleagues [27] demonstrated mostly substantial to excellent inter-rater reliability between online raters with different familiarities of routes in Phoenix using the MAPS Abbreviated Online audit. Wilson and colleagues [18] directly assessed differences in audit site familiarity between in-person and online raters using the Active Neighborhood Checklist and found high overall agreement between in-person raters familiar with the assessment areas (i.e., St. Louis and Indianapolis) and online raters unfamiliar with the assessment areas. It is unknown whether similar agreement would be found using other audit tools, such as MAPS, and in other geographic locations.

### Current study aim

The current study aimed to assess inter-rater reliability between local field raters and online raters with varying degrees of familiarity using an adapted version of the previously-validated MAPS tool [3, 28] across two US regions. Data from San Diego, CA and Phoenix, AZ were used. In-person audits were conducted by raters who resided in their audit region. Raters using the online tool resided in the alternate location, and were unfamiliar with their audit sites. Analyses were conducted for individual-level MAPS items, subscale scores, and overall scores within three sections of the MAPS instrument (i.e., route, street segments, and crossings). High agreement between the site-familiar in-person raters and the site-unfamiliar online raters was hypothesized, thus providing a reliable and less geographically-restricted alternative to in-person audits.

## Methods

### Sample

In-person and online audits were conducted for a total of 120 routes (60 per site) in Phoenix, AZ and San Diego, CA. Phoenix and San Diego are both major US cities with similar population sizes. Phoenix is located in the southwestern US, in the northeastern portion of the Sonoran Desert. San Diego is on the coast of the Pacific Ocean in southern California. The two cities differ in land area, population density, and built environment, as well as climate, topography, and landscaping. Further differentiating the cities is an extensive network of walkable canals interwoven throughout urban Phoenix neighborhoods. Census block groups in both sites were classified according to macro-level walkability and socioeconomic status (SES), using a 2 (high vs. low walkability) × 2 (high vs. low SES) matrix. Walkability was determined using a block group-level composite of net residential density, land use mix, street connectivity and, for San Diego only, retail floor area ratio, as used previously [29]. SES was classified according to Census block group-level median household income.

#### MAPS residential routes

Residential routes (*n* = 40 per site) included a residential origin point and a pre-determined minimum quarter-mile route toward a pre-selected non-residential destination (i.e., cluster of commercial land uses). In Phoenix, ten route origins consisted of randomly selected residential parcels for each SES/walkability quadrant. In San Diego, ten route origins were randomly selected among each SES/walkability quadrant from existing participant households from a previous study [30].

#### MAPS commercial routes

Routes were also pre-selected for 20 commercial clusters near participant households balanced across quadrants in each city (5 clusters × 4 quadrants × 2 cities = total commercial routes, *n* = 40). Commercial cluster was defined as adjacent parcels with three or more commercial land uses. Commercial routes consisted of the street segment in front of a pre-selected commercial cluster and the two crossings on either end.

### Measurement

The MAPS Abbreviated Online tool was adapted from existing instruments [12, 28] to virtually assess the microscale environment for physical activity. The 120-item original [3] and 60-item Abbreviated MAPS [12] field audit tools were validated in four age groups from three US regions. Same method inter-rater reliability was established for the original MAPS using in-person raters [28] and for the online MAPS Abbreviated Online using online raters [27]. The MAPS Abbreviated Online was designed for use with Google Earth, a free geographic software program based on the Google Maps service which displays both satellite and street level images of Earth in high resolution. Google Earth’s Aerial View and Street View platforms offered a perpendicular or oblique view of streets, buildings, and landscapes (Aerial View), as well as eye-level views collecting pedestrian or driver perspectives of streets and buildings via car-mounted 360° cameras (Street View).

Paralleling the original and Abbreviated MAPS in-person instruments, the MAPS Abbreviated Online consisted of four sections: overall route, street segments, crossings, and cul-de-sacs. A total of 62 items were included in the current analyses. Route-level items (35) incorporated characteristics for the full route that were likely consistent (e.g., speed limit, aesthetics) or occurred infrequently (e.g., street amenities, traffic calming) at the segment level. Segment-level items (14) assessed each street segment between crossings on the route (e.g., sidewalks, building heights, road widths and setbacks, street buffers, bicycle facilities, and trees). Crossing items (10) included features of intersections along the route (e.g., crosswalk markings/materials, curb cut presence [i.e., ramps], pedestrian signage and traffic circles). Cul-de-sac items (3) were collected when one or more cul-de-sacs were present within 400 feet of the participant’s home. The cul-de-sacs section assessed the potential recreational environment within a cul-de-sac (e.g., basketball hoops). The number of crossings, segments and cul-de-sacs varied by route, though all routes had at least one segment. Most individual items and subscale scores in the MAPS original [28], Abbreviated in-person [12] and Abbreviated Online [27] tools have demonstrated moderate to excellent inter-rater reliability.

### Data collection

#### Raters and training

Six raters (three per site) were trained and certified using a standard certification process. The training process included ≥15 h of in-person training and a minimum of four test audits (2 residential, 2 commercial) in which raters were required to achieve at least 95% agreement with the expert trainer. For continuous measures, agreement between expert and trainee ratings was defined as plus or minus 1 measurement unit. More details about the training and certification process can be found at [31].

#### In-person audits

In-person route audits were randomly assigned to raters residing in their respective sites (i.e., site-familiar). In-person audits were conducted using the MAPS Abbreviated Online from May to July of 2013 following the standard MAPS field audit protocol detailed here [31].

#### Online audits

Virtual audits replicating the in-person routes were randomly assigned to in-person raters residing in the alternate city (i.e., site-unfamiliar). Aerial and Street View were used to conduct the virtual audits. For Aerial View, assessments were conducted from a zoom-level of approximately 2000 feet above ground level. For Street View, raters virtually traveled the assigned route while rotating perspective 180° approximately every 100 feet and completing the assessment items along the route. Raters used the most recent layer of information on Google Earth and recorded the date of the images. Aerial View image dates for Phoenix ranged from November 2012 to November 2014; Street View images ranged from June 2007 to June 2012. Aerial View images for San Diego were dated March 2013; Street View from December 2008 to July 2013.

#### MAPS abbreviated online scoring

The scoring system applied to online and in-person audits was based on the method conceptualized for the original MAPS [28] and detailed for the MAPS Abbreviated [12] instruments [31]. When multiple crossings, segments and cul-de-sacs subscales were present within the same route, subscales were created using a mean score. Subscale scores within subsections of each instrument section were computed by summing constituent item scores. Subscales were classified according to the direction of expected effect on physical activity and then used to create valence summary scores, explained in detail in [12]. An “overall positive microscale score” was calculated by summing the positive streetscape, aesthetics, segments and crossings scores. Finally, a grand score was calculated by summing the “positive destinations and land use score” and the “overall positive microscale score.” Descriptive statistics for items, subscales, valence, overall and grand scores can be found here: http://sallis.ucsd.edu/Documents/MAPS%20Abb%20Online%20Items_Alt%20Method%20Rel.pdf.

#### Analyses

Dichotomous (no/yes) items from the online MAPS tool were scored as 0/1. Frequency items (0, 1, 2+) were scored as 0, 1, 2. Continuous and descriptive items were recoded as categorical variables for subscales based on distributions, theoretical relevance, and maintaining scoring consistency with other scale items [12, 28]. Inter-rater reliability for subscale, valence and total scores was assessed using intraclass correlation coefficient (ICC). Item-level reliabilities were examined using Kappa statistic (dichotomous variables), ICC (continuous variables) and percent agreement. ICC values were calculated using one-way random effects models with single measurement form (i.e., ICC ((1,1)). Kappa [32] and ICC [33] statistics were evaluated according to guidelines by Landis and Koch [32]: 0.00–0.20 = poor to slight; 0.21–0.40 = fair; 0.41–0.60 = moderate; 0.61–0.80 = substantial; 0.81–1.00 = almost perfect. A limitation to using Kappa and ICC values is sensitivity to low variability in scores, as occurs when there is a very high or low observed occurrence of an attribute. To assist in interpretation of reliability statistics, we also calculated the percentage of audited routes in which the assessed feature was not observed by each rater (i.e., ‘Percent without Feature’). This indicates the potential for adverse influence on Kappa and ICC values due to low variability. For example, the absence of public recreation was noted in 90% of routes in the current sample, resulting in 108 in-person and 109 online routes being coded with scores of 0. Although rater agreement was over 90%, the public recreation subscale ICC was only 0.66, 95% CI [0.55, 0.75]. All data were analyzed using SPSS version 22.0 (SPSS, Inc., Chicago, IL, USA).

## Results

A total of 120 routes with 298 segments, 214 crossings and 18 cul-de-sacs were analyzed. Overall, agreement of individual items between in-person field audits and virtual audits was moderate to near-perfect for approximately 75% of the 120 routes. Item-level descriptive and reliability statistics are provided at http://sallis.ucsd.edu/Documents/MAPS%20Abb%20Online%20Items_Alt%20Method%20Rel.pdf. Subscale, valence and overall scores, scoring components, descriptive statistics and reliability statistics are presented in Tables 1, 2, 3 and 4.

**Table 1:** Route level scales and valence scores (N = 120)

Score name	Possible range	Individual items	Inter-rater agreement (ICC)	Confidence interval (CI)	Rater	Mean	SD	Percent without feature
*Destination and land use:* Positive characteristics								
Residential mix	0–3	0 = commercial 1 = single family 2 = multi-family only and any other mix 3 = apartments over retail only	ICC: 0.73	(0.63, 0.80)	On the ground	1.29	0.69	11.6%
					Online (St view)	1.23	0.65	10.8% commercial
Shops	0–8	Presence of (0, 1, 2+) grocery/supermarket, convenience stores, liquor/alcohol store, retail stores	ICC: 0.86	(0.81, 0.90)	On the ground	1.65	1.93	44.2%
					Online (St view)	1.48	1.78	45%
Restaurant-entertainment	0–8	Presence of (0, 1, 2+) fast food restaurant, sit-down restaurant, café/coffee shop, entertainment	ICC: 0.92	(0.89, 0.95)	On the ground	1.73	1.91	43.3%
					Online (St view)	1.71	1.96	43.3%
Institutional-service	0–6	Presence of (0, 1, 2+) bank, credit union, health-related professional, other service	ICC: 0.85	(0.79, 0.89)	On the ground	1.94	2.03	41.7%
					Online (St view)	2.01	1.89	37.5%
Public recreation	0–2	Presence of (0, 1, 2+) public park	ICC: 0.66	(0.55, 0.75)	On the ground	0.10	0.30	90%
					Online (St view)	0.11	0.36	90.8%
Private recreation	0–2	Presence of (0, 1, 2+) private recreation facilities	ICC: 0.70	(0.59, 0.78)	On the ground	0.21	0.53	85%
					Online (St view)	0.24	0.52	80%
School	0–2	Presence of (0, 1, 2+) school	ICC: 0.53	(0.39, 0.65)	On the ground	0.24	0.52	89.2%
					Online (St view)	0.20	0.50	92.5%
Place of worship	0–2	Presence of (0, 1, 2+) places of worship (e.g., church, synagogue, mosque)	ICC: 0.62	(0.50, 0.72)	On the ground	0.13	0.38	80%
					Online (St view)	0.09	0.34	84.2%
*Destination and land use:* Valence and overall								
Land use positive	0–33	Sum residential mix, shops, restaurant-entertainment, institutional service, public recreation, private recreation, school, place of worship	ICC: 0.93	(0.90, 0.95)	On the ground	7.28	5.91	–
					Online (St view)	7.08	5.50	–
*Streetscape characteristics:* Positive characteristics								
Transit stops	0–2	Number of stops along route (0–6) and amenities at 1st stop (bench, covered shelter), recoded as no stop (0), 1 or more stops (1) or 1 or more stops with amenities at first stop (2)	ICC: 0.95	(0.93, 0.97)	On the ground	0.69	0.93	63.3%
					Online (St view)	0.71	0.94	62.5%
Traffic calming	0–1	Traffic calming characteristics (pedestrian signage, speed humps, curb extensions)	ICC: 0.57	(0.44, 0.68)	On the ground	0.32	0.47	68.3%
					Online (St view)	0.30	0.46	70%
Streetlights	0–1	Presence of street lights (any vs. none)	ICC: 0.91	(0.87, 0.93)	On the ground	0.95	0.22	5%
					Online (St view)	0.96	0.20	4.2%
Driveways	0–1	Presence of less than 6 driveways along route (yes or no)	ICC: 0.87	(0.81, 0.91)	On the ground	0.44	0.50	52.5%
					Online (St view)	0.48	0.50	55.8%
Street amenities	0–4	Presence of street amenities [i.e., trash bins (any vs. none), building overhangs ((any vs. none), bike racks (any vs. none), benches (any vs. none)]	ICC: 0.58	(0.45, 0.69)	On the ground	0.63	0.94	61.7%
					Online (St view)	0.57	0.86	62.5%
*Streetscape characteristics:* Valence and overall								
Streetscape positive	0–9	Transit stops, traffic calming characteristics, street lights, <6 driveways, street amenities	ICC: 0.81	(0.73, 0.86)	On the ground	3.03	1.89	2.5%
					Online (St view)	3.01	1.71	1.7%
*Aesthetics and social characteristics:* Valence and overall								
Aesthetics/social positive	0–3	Buildings well-maintained (100%), no presence of any physical disorder and extent	ICC: 0.15	(−0.03, 0.32)	On the ground	2.25	0.87	3.3%
					Online (St view)	2.09	0.73	0.8%
Aesthetics/social negative	0–2	Buildings not well-maintained (>100%), presence of any physical disorder and extent	ICC: 0.07	(−0.12, 0.24)	On the ground	0.90	0.92	46.6%
					Online (St view)	0.40	0.72	72.5%
Aesthetics/social overall	−2 to 3	Aesthetics/social positive score – aesthetics/social negative scores	ICC: 0.27	(0.10, 0.43)	On the ground	1.38	1.28	17.5%
					Online (St view)	1.70	1.19	8.3%

**Table 2:** Mean of all segments for each route scale and valence scores (n = 120) and cul-de-sac (n = 16) scale and valence scores

Score name	Possible range	Individual items	Inter-rater agreement (ICC)	Confidence interval (CI)	Rater	Mean	(SD)	Percent without feature (%)
*Street segments:* Positive characteristics								
Building height-setback	0–6	Mean of average building height (feet) to average setback (feet) ratio	ICC: 0.56	(0.42, 0.67)	On the ground	1.06	0.47	7.5
					Online (St view)	1.01	0.45	8.3
Building height/road width plus setback ratio	0–3	Mean of average building Height (feet) to Average Road Width (feet) + Average Setback (feet) ratio	ICC: 0.05	(−0.13, 0.23)	On the ground	0.00	0.03	98.3
					Online (St view)	0.02	0.12	95.8
Buffer	0–2	Mean of buffer presence and buffer width	ICC: 0.89	(0.84, 0.92)	On the ground	0.60	0.83	60
					Online (St view)	0.67	0.84	54.2
Bike infrastructure	0–2	Mean of presence of a marked bicycle lane	ICC: 0.85	(0.79, 0.89)	On the ground	0.45	0.76	74.2
					Online (St view)	0.37	0.71	69.2
Trees	0–5	Mean of trees along sidewalk, even or irregular spacing, shade coverage	ICC: 0.66	(0.54, 0.75)	On the ground	1.96	1.01	3.3
					Online (St view)	2.11	0.95	0.8
Sidewalk	0–3	Mean of sidewalk presence, width of sidewalk, sidewalk continuous	ICC: 0.85	(0.79, 0.89)	On the ground	2.77	0.93	6.7
					Online (St view)	2.72	0.94	7.5
Shortcut	0–1	Presence of an informal path (shortcut) connecting to something else	ICC: 0.65	(0.58, 0.71)	On the ground	0.10	0.31	89.6
					Online (St view)	0.08	0.28	91.6
*Street segments*: Valence and overall								
Positive	0–25	Mean of positive subscales (building height-setback, sidewalk, buffers, bike infrastructure, trees, shortcut)	ICC: 0.82	(0.75, 0.87)	On the ground	4.21	1.80	0.8
					Online (St view)	4.26	1.82	1.7

**Table 3:** Crossings—means of all crossings per route: scales and valence scores (N = 107)

Score name	Possible range	Individual items	Inter-rater agreement (ICC)	Confidence interval (CI)	Rater	Mean	(SD)	Percent without feature (%)
*Cul*-*de*-*sacs*								
Overall	0–4	Closeness of cul-de-sac to home, presence of amenities (e.g., basketball hoops), visibility from home	ICC: 0.43	(−0.05, 0.76)	On the ground	−0.05	0.76	1.7
					Online (St view)	−0.10	0.86	0.8
*Crossings:* Positive characteristics								
Crosswalk amenities	0–5	Mean: Presence of crossing aids, marked crosswalk, high-visibility striping, different material than road, curb extensions	ICC: 0.81	(0.74, 0.87)	On the ground	0.54	0.50	32.5
					Online (St view)	0.54	0.50	33.3
Curb quality	0–2	Mean: Presence of pre-crossing and/or post-crossing ramp lined up with crossing	ICC: 0.87	(0.82, 0.91)	On the ground	1.71	0.59	5.8
					Online (St view)	1.67	0.64	7.5
Intersection control	0–3	Mean: Presence of traffic circle, pedestrian walk signals, countdown signals	ICC: 0.92	(0.89, 0.95)	On the ground	0.66	0.76	41.7
					On the ground	0.54	0.49	32.5
*Crossings:* Valence and overall								
Positive	0–10	Mean of positive crossing characteristics scales (amenities, quality, intersection control)	ICC: 0.93	(0.91, 0.95)	On the ground	0.69	0.93	63.3
					Online (St view)	0.71	0.94	62.5

**Table 4:** Final valences and grand scores (n = 120)

Score name	Possible range	Individual items	Inter-rater agreement (ICC)	Confidence intervals (CI)	Rater	Mean	(SD)	Percent without feature
*Grand valence and overall*								
Overall microscale positive	0–47	Sum of 4 subscales: Positive crossing characteristics, positive segments, positive streetscape, positive aesthetics/social	ICC: 0.86	(0.80, 0.90)	On the ground	12.59	4.23	–
					Online (St view)	12.47	3.97	–
Grand score	0–80	Overall microscale positive + land use positive subscale	ICC: 0.93	(0.89, 0.95)	On the ground	20.22	9.08	–
					Online (St view)	19.90	8.46	–

### Route reliability

#### Destinations and land use

Eight positive subscales were analyzed in the destination and land use route section. Seven of eight subscales had substantial to near-perfect agreement, with ICC values ranging from 0.62, 95% CI [0.50, 0.72] (places of worship) to 0.92, 95% CI [0.89, 0.95] (restaurant-entertainment). Moderate agreement was found for the schools subscale (ICC = 0.53, 95% CI [0.39, 0.65]). All five destinations and land use subscales with ICC values <0.70 (residential mix, places of worship, schools, private and public recreation) were infrequent occurrences across routes, which resulted in values of zero for more than 80% of audits. The positive valence score created by summing the eight positive subscales showed near-perfect agreement between site-familiar in-person and site-unfamiliar online raters (ICC = 0.93, 95% CI [0.90, 0.95]). Results for route reliability subscales and valence scores are presented in Table 1.

#### Streetscape characteristics

A positive subscale score for streetscape characteristics was comprised of five items/subscales. Near perfect inter-rater reliability was found for transit stops (ICC = 0.95, 95% CI [0.93, 0.97]), streetlight presence (ICC = 0.91, 95% CI [0.87, 0.93]) and driveway presence (ICC = 0.87, 95% CI [0.81, 0.91]). Presence of traffic calming characteristics and street amenities both had moderate agreement, with ICC values of 0.57, 95% CI [0.44, 0.68] and 0.58, 95% CI [0.45, 0.69] respectively. The positive valence subscale score demonstrated near-perfect agreement (ICC = 0.81, 95% CI [0.73, 0.86]). Results for streetscape characteristics are presented in Table 1.

#### Aesthetics and social characteristics

Positive and negative valence scores and an overall subsection score (positive minus negative valence scores) were computed for the aesthetics and social characteristics items. Constituent items assessed the maintenance and disorder of the environment. Similar to the poor to fair agreement found at the item level (see http://sallis.ucsd.edu/Documents/MAPS%20Abb%20Online%20Items_Alt%20Method%20Rel.pdf), agreement was poor to slight for both the positive and negative valence scores (ICCs = 0.15, 95% CI [−0.03, 0.32] and 0.07, 95% CI [−0.12, 0.24], respectively), and fair for the overall aesthetics and social characteristic score (ICC = 0.27, 95% CI [0.10, 0.43]). Results for aesthetics and social characteristics are presented in Table 1.

#### Segment reliability

Six positive subscales in the street segments section were evaluated (Table 2). Four subscales had substantial to near-perfect agreement, with ICC values ranging from 0.66, 95% CI [0.54, 0.75] (trees) to 0.89, 95% CI [0.84, 0.92] (buffers). Moderate agreement was found for the building height setback subscale (ICC = 0.56, 95% CI [0.42, 0.67]). The ICC value for the building height to road width plus setback ratio subscale was low (ICC = 0.05, 95% CI [−0.13, 0.23]). However, it should be noted that over 95% of the calculated ratios were zero for both online and in-person audits, helping explain the poor ICC. The segments positive valence score had near-perfect inter-rater agreement (ICC = 0.82, 95% CI [0.75, 0.87]).

#### Crossing reliability

Three positive subscale scores were evaluated in the crossings section (Table 3). All three had near-perfect inter-rater agreement, with ICCs ranging from 0.81, 95% CI [0.74, 0.87] (crosswalk amenities) to 0.92, 95% CI [0.89, 0.95] (intersection control). Near-perfect agreement was also found for the crossings positive valence score (ICC = 0.93, 95% CI [0.91, 0.95]).

#### Cul-de-sac reliability

One positive valence score analyzed for the cul-de-sacs section demonstrated moderate agreement between in-person field audits and virtual audits (ICC = 0.43, 95% CI [−0.05, 0.76]) (Table 3). It is not included in the overall positive microscale score or the grand score because its relation to physical activity is unclear.

#### Overall positive microscale and grand scores reliability

An overall positive microscale score was calculated by summing positive valence scores for routes (streetscape characteristics and aesthetics and social characteristics), segments, and crossings. Agreement was near-perfect between in-person and online auditors (ICC = 0.86, 95% CI [0.80, 0.90]). Similarly, the grand score, created by adding the positive valence score for destinations and land use to the overall positive microscale score, had near-perfect inter-rater agreement (ICC = 0.93, 95% CI [0.89, 0.95]). Results are presented in Table 4.

## Discussion

The present study examined alternate-form reliability between in-person and online data collection methods using the MAPS Abbreviated audit tool. Overall, there was substantial to near-perfect agreement between site-familiar in-person raters and site-unfamiliar online raters for 21 of the 30 subscales, valence and subsection scores. Lowest reliability was found with aesthetics and social disorder scales. Inter-rater agreement was also near-perfect for the total positive microscale and grand summary scores. These results indicated that microscale environmental features supporting physical activity can be reliably assessed virtually using the MAPS online tool.

The present study was designed to address the question of whether raters auditing built environment features with the MAPS Abbreviated measure need to be personally familiar with a city or region. Virtual raters unfamiliar with an environment could differ in degree of understanding or familiarity with a city or its particular built environment features [25, 26]. If online auditing by raters not familiar with the region is supported, centralized auditing could replace expensive in-person audits for reliable features. Because previous studies used local raters or did not specify whether raters were familiar with an area, it was uncertain whether raters’ familiarity influenced agreement. In the current study, agreement was generally near-perfect to moderate between in-person and virtual raters for the majority of items and scales. These results are consistent with Wilson et al. [18] who found high agreement between in-person and site-unfamiliar online auditors in two mid-western cities using another audit tool. Both studies provided consistent evidence using different audit tools that centralized virtual audits can be used without regard to auditors’ physical locations or familiarities with audit areas in the US.

MAPS Online subscales with the highest agreement (e.g., land use and destinations, transit stops, streetlights, and intersection controls) had several common qualities. Constituent items in these subscales were generally more quantitative than qualitative (e.g., presence/absence of street buffers and buffer width). Attributes were usually large or easily distinguishable (e.g., sidewalks, traffic circles, pedestrian walk signals) and were likely to remain stable over time. These results are consistent with previous comparisons of online and in-person audits which similarly found highest agreement for objectively-assessed items that were relatively impervious to time effects [21] and were highly visible, regardless of audit method [22, 23, 34]. These results suggest that site-unfamiliar and site-familiar online raters performed similarly for the majority of microscale features.

Similar to other studies [17, 21, 34, 35], cross-method agreement was lowest for the aesthetics and social characteristics subsection (e.g., presence and extent of graffiti or condition of facades). Constituent items in this subsection generally had low kappa values and percent agreement. The only exception was the presence of softscape features (e.g., any landscaping), which had 94.2% agreement. Although the kappa value was low for this item (k = −0.01), this was likely due to the high prevalence of softscapes observed along routes for both rating methods (114/120 in-person, 119/120 online) limiting variance in this item.

It is possible that maintenance and disorder items are more difficult to virtually assess when unfamiliar with an area. Online images likely do not provide the same degree of environmental context as physically walking a route, making qualitative assessments difficult. In addition, some of these indicators may be difficult to see on Google Street View or views may be temporarily or permanently blocked. Maintenance and disorder items are also more susceptible to temporal variability than built environment features for streetscape audits in general (e.g., shade or presence of litter or graffiti vs. presence of street lights), meaning that observed conditions may vary considerably between any two time points. Thus, time lapses between online image collection dates and in-person audits may have resulted in inconsistent rater observations. Despite the low agreement for aesthetics and social characteristics items, the MAPS Abbreviated Online tool includes these items to be consistent with the in-person MAPS Abbreviated tool. Data collected online may still be a useful indicator of overall maintenance and disorder, though with limited precision. It is likely that when online raters can notice graffiti and other signs of disorder using Street View, the extent of the disorder may be substantial and worth differentiating from especially well-maintained neighborhoods. However, some investigators using MAPS Abbreviated online may want to exclude these items from data collection, summary scores, or analyses, due to low reliability.

### Strengths and limitations

This study had notable strengths that contribute to the generalizability of findings. First, the sample size was large, consisting of 120 routes with 298 street segments and 214 crossings in two geographically distinct metropolitan areas (i.e., coastal city and desert mountain city). Similar studies evaluating reliability between in-person and virtual audits have had small sample sizes [13, 15, 17, 20], limited audit neighborhoods to a single metropolitan area [13–15, 20] or been restricted in regional diversity [18]. Additionally, a route selection protocol was used to approximate the built environment as individuals would encounter it in everyday life.

The current study’s sampling design equally distributed route origins among neighborhoods with high and low SES and walkability. While several prior studies accounted for neighborhood SES [15, 18], we are not aware of any that also controlled for the influence of macro-level walkability (i.e., using GIS data). The inclusion of diverse neighborhoods also likely maximized the prevalence and variance in audit features. Present results suggested online microscale audits were reliable regardless of neighborhood SES, and in neighborhoods that varied on other macro-level features contributing to walkability [29]. It has been suggested that item reliabilities may differ by location [36], in part due to differences in the prevalence of audit features across sites [36, 37] or low variance in audit answers [34]. Therefore, direct reliability comparisons between sites are difficult because low reliability coefficients may be more indicative of frequency of occurrence than differences in agreement. To overcome this limitation, the present study maximized the prevalence and variability of audit features by combining data collected in two cities. Because each site was not analyzed separately, it is possible that the reliability of some items differed by site. It would be important to disentangle prevalence effects from poor rater agreement to understand whether some items are perceived differently depending on location. This may be addressed in future work by increasing the number and diversity of sampled sites and neighborhoods to ensure sufficient variance in item scores across locations.

Several limitations in the current study were inherent to virtual audits in general. First, auditors were reliant on the images available through Google Maps, which dated as far back as 2007 and possibly did not accurately depict the streetscape at the time of the in-person audits. Differences in the timing of in-person and online data collection could have been a potential source of bias. It is possible that some streetscape characteristics were assessed differently depending on what time of day or year data collection occurred. For instance, raters in Phoenix may have differentially assessed the types of landscape depending on whether images were taken during exceptionally hot and dry desert summer months or during the more temperate winter or spring blooming season. Likewise, features may have been assessed differently depending on when data were collected relative to the time of day of litter/trash removal, pedestrian and automobile activity, or weather occurrence.

Additionally, image dates varied within and across audit areas, as well as between different image views for the same areas. For example, Google Street View image dates in the present study ranged from 2007 to 2012 for Phoenix and 2008–2013 for San Diego. Aerial Views (based on satellite imagery) were more recent, ranging from 2012 to late 2014. This temporal difference in views occasionally resulted in image inconsistencies, such as a more recent Aerial View displaying a crosswalk marked with high-visibility striping that appeared as an unmarked crosswalk in older Street View images.

A limitation of Street View was that the images were not always complete for an entire segment, such that there were occasionally missing street sections or intentionally obscured image areas for privacy reasons at the request of a home or business owner. However, these issues occurred infrequently and were noted. Relatedly, sometimes images contained large busses, trucks, foliage or other objects that obstructed views along a segment. Along with image composition, virtual audits were reliant on image resolutions that provided a sufficient level of detail for assessed characteristics. Fine-grained attributes such as trip hazards on sidewalks, which performed poorly in the present study, may have been difficult to discern with available image resolutions.

Based on current and previous findings, microscale aesthetics and social features appeared difficult to assess reliably using currently available virtual tools. It is possible these qualities may be better assessed using alternative data collection methods. Future work may explore opportunities that take advantage of emerging technologies to characterize microscale attributes that are highly subjective and transient in nature. For example, recently-developed geolocation-aware mobile crowdsourcing technology could be used in conjunction with measurement burst designs [38] to collect repeated sequences of perceptual data over different time scales. Such data may complement existing online audit tools to facilitate a better understanding about the temporal dynamics of the perceived environment, and if or how this relates to physical activity behaviors.

Most of the challenges noted above occurred infrequently in the present study and given the high degree of overall agreement, did not seem to adversely affect the reliability and practicality of using the MAPS Abbreviated Online tool. Some limitations may be mitigated by establishing rules that would become part of virtual rater training. For example, a rule could be implemented to use features seen in aerial and Street Views based on the most relevant date or date closest to the time period of interest. If the most recent images are desired, researchers may choose to plan audit timelines around anticipated time frames for Street View image collection, published for each district within specified regions in each country [39]. Because Google Street View now provides historical images, virtual audits may be conducted retrospectively or longitudinally. This offers the potential to learn more about how features change over time in a way that would not be feasible with field audit methods. However, researchers are limited to the historical images available, which may not necessarily correspond to time periods of interest in all locations.

### Implications for applications of MAPS

Results from the present study support the MAPS Abbreviated Online audit tool as a reliable alternative to in-person audits generalizable to similar US cities. From an international standpoint, findings may also extend to comparable mountain, desert or coastal metropolitan areas with relatively warm dry climates. Results from Vanwolleghem and colleagues [34] support the use of MAPS outside of the US. Acceptable inter-rater reliability was found for the majority of items assessed in-person, online and using alternate methods in Belgium using the MAPS Global tool, which was adapted for international use [34]. Similar work is being conducted in several other international locations to ascertain the generalizability of MAPS Global outside of the US.

Among the advantages to implementing the MAPS Abbreviated Online tool in research settings are elimination of travel time and costs, weather-related concerns and potential threats to personal safety associated with in-person audits. Moreover, it would enable centralized audit operations, facilitating procedural standardization and efficient use of personnel time. Conceivably, online auditing could promote growth in the study of microscale environmental influences on physical activity by increasing the number, scale and geographic diversity of investigations, with data collected from the same validated instrument. Thus, future studies would benefit from validating MAPS online scores with physical activity data collected from participants in diverse geographic regions and neighborhood types.

## Conclusions and recommendations

Audits conducted using publically available online tools appear to be safe, convenient, efficient and cost-effective alternatives to in-person field audits, even with auditors unfamiliar with the assessed regions. Virtual audits are less resource-intensive than in-person audits and are unrestricted by geographic proximity to the audit location or weather. At the time of writing, Google Street View images were available for over 250 regions/states in more than 25 countries, and are expected to continue expanding into areas not currently covered [39]. The development and validation of reliable online audit tools, such as MAPS Abbreviated Online, can provide a means for understanding microscale features in increasingly diverse environments as more locations become virtually accessible.

## Abbreviations

AZ: Arizona
CA: California
GIS: geographic information system
ICC: intraclass correlation coefficient
K: kappa
MAPS: Microscale Audit of Pedestrian Streetscapes
SES: socioeconomic status
US: United States
